# Epithelial cell-cell interactions in an overcrowded environment: jamming or live cell extrusion

**DOI:** 10.1186/s13036-024-00442-3

**Published:** 2024-09-05

**Authors:** Ivana Pajic-Lijakovic, Milan Milivojevic, Peter V. E. McClintock

**Affiliations:** 1 Faculty of Technology and Metallurgy, Department of Chemical Engineering, University of Belgrade, Belgrade, Serbia; 2 Department of Physics, Lancaster University, Lancaster, LA1 4YB UK

**Keywords:** Cell-cell interactions, Overcrowded environment, Collective cell migration, The Marangoni effects, Viscoelasticity

## Abstract

Epithelial tissues respond strongly to the mechanical stress caused by collective cell migration and are able to regulate it, which is important for biological processes such as morphogenesis, wound healing, and suppression of the spread of cancer. Compressive, tensional, and shear stress components are produced in cells when epithelial monolayers on substrate matrices are actively or passively wetted or de-wetted. Increased compressive stress on cells leads to enhanced cell-cell interactions by increasing the frequency of change the cell-cell distances, triggering various signalling pathways within the cells. This can ultimately lead either to cell jamming or to the extrusion of live cells. Despite extensive research in this field, it remains unclear how cells decide whether to jam, or to extrude a cell or cells, and how cells can reduce the compressive mechanical stress. Live cell extrusion from the overcrowded regions of the monolayers is associated with the presence of topological defects of cell alignment, induced by an interplay between the cell compressive and shear stress components. These topological defects stimulate cell re-alignment, as a part of the cells’ tendency to re-establish an ordered trend of cell migration, by intensifying the glancing interactions in overcrowded regions. In addition to individual cell extrusion, collective cell extrusion has also been documented during monolayer active de-wetting, depending on the cell type, matrix stiffness, and boundary conditions. Cell jamming has been discussed in the context of the cells’ contact inhibition of locomotion caused by cell head-on interactions. Since cell-cell interactions play a crucial role in cell rearrangement in an overcrowded environment, this review is focused on physical aspects of these interactions in order to stimulate further biological research in the field.

## Introduction

Epithelial tissues respond strongly to internal and external mechanical stresses via dynamic cellular rearrangements [1–3]. During such processes, cells are able to self-regulate the magnitude of mechanical stress and the corresponding cell packing density [3–5]. Movement of epithelial collectives induces the generation of cell mechanical stress, both normal (compressive or tensional) and shear [1, 6, 7]. While the accumulation of normal stress causes a change in cell packing density, the cell packing density is retained under cell shear stress. Cell tensional stress leads to a decrease, while cell compressive stress causes an increase in cell packing density by influencing the cell-cell interactions. Cell shear stress has no impact on cell packing density, but influences the strength of cell-cell and cell-matrix adhesion contacts [8]. All stress components, generated during collective migration of epithelial monolayers, have been experimentally confirmed [1, 2, 6]. The anisotropic nature of collective cell migration implies that cell monolayer extension in one direction, caused by cell movement, induces compression in the other in order to maintain tissue structural integrity [1, 9, 10].

The concept of active wetting, caused by collective cell migration [11], can be interpreted as active extension in the direction of cell movement and passive compression in the perpendicular direction. Pérez-González et al. [11] pointed out that active wetting results from the competition between traction forces and contractile intercellular forces. The opposite case, in the form of active de-wetting, has been observed [11]. Both processes, active wetting/de-wetting, show oscillatory behaviour caused by interplay between physical parameters such as: mechanical stress, epithelial surface tension, matrix surface tension, and epithelial-matrix interfacial tension [1, 12]. The epithelial surface tension represents the surface tension between multicellular surface and surrounding liquid medium. Whether epithelial tissue undergoes active wetting or de-wetting depends on the inter-relation between specific adhesion and cohesion energies [12]. The cohesion energy of cell monolayers depends on the strength of cell-cell adhesion contacts and cell contractility and correlates with epithelial surface tension [13]. The cell-matrix adhesion energy depends on the strength of cell-matrix adhesion contact, i.e. focal adhesions (FAs) [14]. A special interest here is to discuss the cell response to compressive stress within migrating epithelial monolayers.

An increase in the cell compressive mechanical stress, caused by collective cell migration, can trigger the cell jamming state transition and/or live cell extrusion. Both processes (cell jamming/unjamming transitions and cell extrusion) provide ways by which epithelium regulates the cell compressive stress corresponding to the cell packing density [3–5]. Despite the fact that both cellular processes have been widely studied, it is not clear which of them is preferred in an overcrowded environment caused by higher compressive mechanical stress. An increase in the compressive stress, due to tighter cell packing, stimulates cell-cell orientational interactions, of which two types need to be taken into consideration: (1) cell head-on interactions and (2) cell glancing interactions. The head-on interactions cause contact inhibition of locomotion, i.e. cell re-polarisation by inducing weakening of cell-cell and cell-matrix adhesion (focal adhesions FAs) contacts and a change in the direction of cell movement [15, 16]. This type of cell-cell interaction has been recognised as a possible cause of the cell jamming state transition [15–17]. Cell jamming occurs when the time between two head-on interactions is shorter than the cell re-polarisation time [18]. In this case, cells undergo a contractile-to-non contractile cell state transition by reinforcing the strength of FAs [19]. It would be interesting to discuss the underlying mechanism by which jamming multicellular domains induce a decrease in the cell packing density. The other cellular process that can be activated under higher cell packing density is live cell extrusion.

In contrast to cells jamming, cells retain their contractile state during live cell extrusion. The targeted cell (or cells) lose their FAs. Cell extrusion actively regulates development and facilitates homeostasis by expelling cells from overcrowded regions [4]. Although many of the molecular factors involved in cell extrusion are known, little is known about the mechanical basis of cell extrusion, and it is not clear which physical factor is directly responsive to the cell’s loss of FAs. Saw et al. [5] pointed out that the generation of topological defects in cell alignment within cell monolayers is a prerequisite of cell extrusion. These topological defects could be related to cell glancing interactions. Glancing interactions are a form of cell orientational interaction that occurs when cells adjust their alignment towards the direction of collective cell migration. Perturbation of cell alignment leads to an imbalance of the intercellular forces responsible for cell realignment ultimately resulting in the re-establishment of force balance [5, 20]. These interactions are not strong enough as head-on interactions and cannot induce cell re-polarisation and consequently weakening of cell-cell adhesion contacts, but they can cause weakening of FAs similarly to cell head-on interactions. While cell head-on interactions have been widely studied in the context of the inhibition of locomotion by cell contact [15, 16], the role of cell glancing interactions in the weakening of FAs leading to cell extrusion has not yet been examined.

The main goal of this theoretical consideration is to discuss the role of cell shear and compressive stress components in the generation of topological defects within epithelial monolayers and to identify the physical factors responsible for: (1) the cell jamming/unjamming transitions; (2) live cell extrusion; and (3) epithelial de-wetting, which leads to the formation of a 3D rim-like cellular structure which can be considered as collective live cell extrusion. The physical mechanisms underlying cell-cell interactions will also be discussed in the context of the phenomena considered.

## Active wetting/de-wetting of epithelial monolayers

Epithelial monolayers undergo active/passive wetting/de-wetting, depending on whether the monolayer displacement occurs via collective cell migration, or not. The wetting or de-wetting that occurs through collective cell migration is considered as an active process. However, cell displacement can be driven by the gradient of the epithelial-matrix interfacial tension in the direction perpendicular to cell movement corresponding to the passive process. Another example of passive de-wetting is the rearrangement of epithelial monolayers on non-adhesive substrate matrix, which suppresses cell movement [21]. Epithelial wetting/de-wetting depends on the interplay between specific adhesion and cohesion energies in the form of the spreading factor $\:{S}^{e}\left(r,\tau\:\right)={\omega\:}_{a}-{\omega\:}_{c}$, where $\:r$ is the local coordinate, $\:\tau\:$ is the long-time of hours, $\:{\omega\:}_{a}\left(r,\tau\:\right)$ is the specific cell-matrix adhesion energy, and $\:{\omega\:}_{c}\left(r,\tau\:\right)$ is the specific cohesion energy. The cell-matrix adhesion energy depends on the strength of cell-matrix adhesion contacts and has been expressed as: $\:{\omega\:}_{a}\left(r,\tau\:\right)={\rho\:}_{a}\frac{1}{2}{k}_{FA}{\left|{\overrightarrow{\varvec{u}}}_{\varvec{m}}\right|}^{2}$ where $\:{\rho\:}_{a}$ is the surface density of cell-substrate adhesion contacts, $\:{k}_{FA}$ is the elastic constant of single cell-substrate adhesion contacts (FA), and $\:{\overrightarrow{\varvec{u}}}_{\varvec{m}}$ is the matrix displacement field [14]. The strength of the cell-matrix adhesion contacts depends on: changes in cytoskeletal tension [22], conformational changes of vinculin (a cytoplasmic actin-binding protein) [23], rearrangement of microtubules [24], and inter- and intra-chain interactions of integrin filaments within the FA [25]. The rearrangement of FA depends on the rheological behaviour of the matrix and its stiffness [26]. The cohesion energy can be expressed as: $\:{\omega\:}_{c}\left(r,\tau\:\right)=2{\gamma\:}_{e}\left(r,\tau\:\right)$ where $\:{\gamma\:}_{e}\left(r,\tau\:\right)$ is the epithelial surface tension. Epithelial cells undergo wetting when the spreading factor $\:{S}^{e}\left(r,\tau\:\right)>0$ and de-wetting when $\:{S}^{e}\left(r,\tau\:\right)<0$. Specific cohesion energy is the energy required to separate a multicellular system into two parts by creating two homotypic multicellular surfaces.

The epithelial surface tension $\:{\gamma\:}_{e}$ represents the change in surface energy of a single multicellular surface $\:{E}_{S}$ by changing the surface area, and can be expressed as: $\:{\gamma\:}_{e}=\frac{\partial\:{E}_{S}}{\partial\:A}$ (where $\:A$ is the surface area). The surface energy can be expressed as: $\:{E}_{S}=\sum_{i}^{}\frac{K}{2}{\left({A}_{ceffi}-{A}_{0}\right)}^{2}+\sum_{i,j}^{}\varLambda\:{l}_{ij}+\sum_{i}^{}\frac{{T}_{con\:i}}{2}{{L}_{i}}^{2}$ (where $\:{A}_{ceffi}$ is the effective surface area of the i-th cell, $\:K$ is the effective modulus of the cell around its preferred surface area $\:{A}_{0}$, $\:{l}_{ij}$ is the interface length between the i-the and j-th cells, $\:\varLambda\:$ is the line tension per unit interface length between two cells, $\:{T}_{con\:i}$ is the contractility coefficient, and $\:{L}_{i}$ is the perimeter of the i-th cell) [27]. Epithelial surface tension depends on the strength of mediated cell-cell adhesion contacts, cell contractility, and monolayer extension/compression [13, 27, 28]. During their collective migration, cells use Adherens Junctions (AJs) to couple mechanically and as an important source of signalling that coordinates collective behaviour [29]. Ones of the main components of AJs are proteins from the cadherin family. Cadherins are transmembrane glycoproteins containing an extracellular domain that mediates cell-cell adhesion via homophilic or heterophilic interactions and an intracellular domain that controls signalling cascades involved in a variety of cellular processes, including polarity, gene expression, etc. Cell contractility enhances the strength of E-cadherin-mediated cell-cell adhesion contacts [13]. Consequently, the surface tension of active, contractile cells is higher than the surface tension of non-contractile ones, i.e., $\:{\gamma\:}_{e}^{m}>{\gamma\:}_{e}^{r}$, where $\:{\gamma\:}_{e}^{m}$ and $\:{\gamma\:}_{e}^{r}$ are the epithelial surface tensions of contractile (migrating) and non-contractile (resting) cells, respectively. Extension of cell monolayers, caused by cell wetting or an externally-induced force, also causes an increase in the epithelial surface tension [10, 27]. However, compression can induce weakening of the cell-cell adhesion contacts caused by cell-cell interactions [15]. These interactions can lead to the contact inhibition of locomotion, resulting in a decrease of the epithelial surface tension [28, 29]. This decrease, accompanied by the cohesion energy of monolayers, stimulates the cell wetting.

The main characteristics of migrating epithelial monolayers are their inhomogeneous distribution and long-term oscillations of the following physical parameters: the cell packing density, the cell velocity, the corresponding strain, the cell mechanical stress, the epithelial surface tension, and the cell-matrix adhesion energy, resulting in an inhomogeneous distribution of the epithelial spreading factor [1, 2, 30]. The inhomogeneous distributions of cell packing density, cell velocity, and stress-strain have been confirmed experimentally by Serra-Picamal et al. [1], Notbohm et al. [2], and Tlili et al. [30]. The inhomogeneous distribution of the strength of cell-cell adhesion contacts, which causes the inhomogeneous distribution of epithelial surface tension, has been measured by Pérez-González et al. [11]. An inhomogeneous distribution of the concentration of collagen fibers caused by cell tractions, pointing to the inhomogeneous matrix surface tension, has been measured by Clark et al. [26]. Notbohm et al. [2] discussed the anisotropic nature of cell mechanical stress caused by collective cell migration. Moreover, the epithelial monolayer can be treated as an ensemble of multicellular domains characterised by a constant epithelial spreading factor within each domain. The domain represents a group of cells described by homogeneous distributions of: (1) cell speed [31], (2) cell packing density [29, 32], (3) properties of movement such as coordination and cooperation [33], and (4) the corresponding viscoelastic behavior [18]. The coordination of a cell group is identified with directional alignment, while the cooperation between cells within the group depends on the properties of cell-cell interconnectivity. These multicellular domains are formed as a result of the cells’ tendency to establish an ordered trend of cell migration. They persist for some period of time before disappearing again as a consequence of cell-cell interactions. The inhomogeneity of the spreading factor induces various scenarios of cell wetting/de-wetting.

Douezan and Brochard-Wyart [21] considered passive de-wetting of murine sarcoma (S-180) cell monolayers on a non-adhesive substrate matrix. This cell line is transfected to express E-cadherin molecules at their surface and form cohesive multicellular systems with a surface tension similar to that of epithelial collectives. The spreading factor, in this case, is $\:{S}^{e}\left(r,\tau\:\right)\approx\:-{\omega\:}_{c}$, while the cell-matrix adhesion energy is $\:{\omega\:}_{a}=0$. These cell monolayers perform inhomogeneous passive de-wetting by leaving cell-free areas on the surface and by forming three-dimensional cellular structures. The passive de-wetting is induced by work of the epithelial surface tension in decreasing in the monolayer surface area, while cells cannot establish focal adhesions and consequently, cannot migrate. Frictional effects between the cell monolayer and substrate matrix, which depend on the matrix rigidity, have a feedback impact on the rate of the monolayer compression caused by the de-wetting. This, in turn affects the monolayer cohesiveness and viscoelasticity. An increase in the monolayer cohesiveness and the rigidity of the substrate matrix reduces the formation of holes [21]. An increase in the concentration of E-cadherin molecules per unit area of cell surface and the rigidity of the matrix cause: (1) an increase in the epithelial surface tension and (2) the establishment of a more homogeneous distribution of epithelial surface tension, which consequently reduces the formation of holes. In contrast to the previous scenario where cells undergo de-wetting on a non-adhesive substrate, the migration of cells on an adhesive substrate leads to the formation of focal adhesions with the matrix. In this particular case, cells can diminish the frictional effects between the cell and the matrix by remodelling the focal adhesions and subsequent attachment and detachment. In this case, the matrix stiffness has an impact on both cell-cell and cell-matrix adhesion contacts [33].

While cell monolayers undergo passive de-wetting on non-adhesive substrates, these monolayers undergo active wetting/de-wetting on adhesive substrates. Pérez-González et al. [11] considered active wetting/de-wetting of human breast adenocarcinoma cells (MDA-MB-231) forming monolayers on a collagen-coated substrate. The MDA-MB-231 cells were transfected with a dexamethasone-inducible vector containing the human E-cadherin coding sequence. Cell monolayers performed active wetting within 24 h and then underwent active de-wetting within the next 36 h by forming a 3D rim-like cell structure. The wetting (extension) of cell monolayers causes an increase in the epithelial surface tension, resulting in a decrease in the spreading factor. When the spreading factor became $\:{S}^{e}\left(r,\tau\:\right)<0$ cells underwent de-wetting. The epithelial surface tension and the concentration of E-cadherin, oscillates around the equilibrium value during de-wetting [11]. An increase in cell packing density within the peripheral region of cell monolayers during wetting induces the formation of 3D cell structure in the form of a rim during de-wetting [11]. Similar 3D cell structure was observed during rearrangement of confluent epithelial Madin Darby Canine Kidney (MDCK) cell monolayers within a circularly bounded adhesive substrate [34]. The phenomenon has been discussed in the context of collective live cell extrusion [11, 34]. Pérez-González et al. [11] measured 2D tensional stress during the monolayer de-wetting. However, tension in one direction induces compression in the other in order to maintain the monolayer’s structural integrity. The inter-relation between compressive and extensional strains responsible for the generation of the corresponding normal stress components $\:{\sigma\:}_{cxx}$ and $\:{\sigma\:}_{cyy}$ is: $\:{\epsilon\:}_{xx}=-\nu\:{\epsilon\:}_{yy}$ (where $\:\nu\:$ is the Poisson’s ratio which satisfies the condition $\:\nu\:\ne\:0.5$). It is consistent with multicellular systems being compressible. The fusion of two confluent skin fibroblast cell aggregates caused a decrease in the volume of two-aggregate systems by a factor of 2.38× within 140 h [35]. A cancer cell spheroid of CT26 cells lost 15% of its volume under an osmotic stress of 5 kPa, while the cell volumes were approximately constant [36]. The change of cell packing density during uni-axial extension depends on the magnitude of the Poisson’s ratio $\:\nu\:$. The cell packing density can: (1) decrease for $\:\nu\:<0.5$, (2) stay constant for $\:\nu\:=0.5$ (an isotropic behaviour), or (3) increase for $\:\nu\:>0.5$. Moisdon et al. [37] revealed that the experimental value of the Poisson’s ratio of MDCK epithelial monolayers is $\:0.77\pm\:0.01$. Tambe et al. [6] analyzed the impact of change in the Poisson’s ratio on cell stress distribution caused by collective cell migration for $\:\nu\:\le\:0.5$ without measuring this parameter.

Serra-Picamal et al. [1] considered the active wetting of MDCK cell monolayers on a collagen-coated substrate matrix over an interval of 7 h. These cells undergo inhomogeneous wetting, such that those located in the central region of the monolayers were less active in comparison with those located in the peripheral region. Only locally active de-wetting was observed, taking the form of local cell backwards flows. Collisions of forwards and backwards flows can induce the accumulation of compressive stress. Serra-Picamal et al. [1] observed oscillations of the tensional stress in the *x*-direction $\:{\sigma\:}_{cxx}$. The stress change in the *y*-direction $\:{\sigma\:}_{cyy}$ was not measured. However, extension of the monolayer in the *x*-direction has to induce compression in *y*-direction, to some extent, in order to maintain the monolayer’s structural integrity. Shear stress $\:{\sigma\:}_{cxy}$ is also generated during the wetting of MDCK cell monolayers [1]. Global de-wetting was not observed within 7 h. This left open the question: Can the de-wetting occur within a longer time-period, or not? Pérez-González et al. [11] observed the occurrence of de-wetting 24 h after the extension (wetting) of cell monolayers. Tlili et al. [30] pointed out that free expansion (i.e. active wetting) of MDCK monolayers caused an inhomogeneous distribution of cell packing density. Some multicellular domains reached confluence, while others underwent cell jamming. The main characteristic of cell rearrangement within confluent cell domains is long-term oscillations of cell compressive stress [2].

In the next section, the generation of mechanical stress and its impact on the formation of topological defects during epithelial monolayer wetting/de-wetting will be discussed.

## Cell mechanical stress generation caused by cell wetting/de-wetting

The cell normal (tensional/compressive) stress is responsible for changes in the cell packing density, while the shear stress has no impact on the cell packing density. Cell tensional stress causes a decrease in the cell packing density, while compressive stress induces an increase in the cell packing density over a time-scale of hours. An increase in cell packing density intensifies cell-cell interactions, which have a feedback impact on cell cohesion and adhesion energies. The probability of a cell-cell interaction is proportional to the cell volume fraction φ. Cell mechanical stress is caused by in-plane cell strain generated during epithelial monolayer inhomogeneous wetting/de-wetting. The in-plane cell strain can be uni-axial or biaxial depending on cell-cell interactions. Consequently, cell compressive, tensional, and shear stress components are generated during cell rearrangement as follows:

- When a cell monolayer undergoes anisotropic active wetting, then extension in the direction of cell movement (i.e., active wetting) leads to compression in the direction perpendicular to cell movement (i.e., passive de-wetting) in order to maintain the monolayer’s structural integrity.
- Anisotropic compression in the direction of cell movement (i.e., active de-wetting) results in a generation of cell tensional stress in the direction perpendicular to the cell movement (i.e., passive wetting).
- Some multicellular domains perform more intensive wetting than surrounding domains and compress them. Pérez-González et al. [11] confirmed this experimentally. The wetting of the monolayer’s central region was more intensive than the wetting of its peripheral region, caused by the radial distribution of epithelial surface tension accompanied by a concentration of E-cadherin [11].
- Some multicellular domains undergo active wetting, while others undergo active de-wetting. The consequence of the existence of the wetting and de-wetting domains can be the generation of forwards and backwards flows [1]. Collisions between these flows can generate additional compressive stress.
- Some multicellular domains undergo inhomogeneous de-wetting, which causes an inhomogeneous accumulation of the cell compressive stress and can induce the formation of holes within the monolayer [21].
- Cell shear stress can be generated along the borders between multicellular domains depending on their velocities. When the cell packing density is lower than or equal to $\:{n}_{conf}$ (where $\:{n}_{conf}$ is the cell packing density in the confluent state), local cell shear stress generation has been recorded within wetting MDCK cell monolayers [1, 6].

These scenarios demonstrate that cell compressive stress can be accumulated locally even when the epithelial monolayers undergo wetting, while the de-wetting of the monolayers results in an intense accumulation of compressive stress. It is consistent with the experimental observation of cell jamming domains as an indicator of cell compressive stress, by Serra-Picamal et al. [1], Nnetu et al. [32], Tlili et al. [30], and by many others who have considered active wetting of epithelial monolayers. The maximum compressive stress generated during the rearrangement of confluent MDCK cell monolayers, and the maximum tensional stress caused by the wetting of MDCK cell monolayers, were 300 Pa [1, 2]. The cell shear stress generated during the wetting of MDCK epithelial monolayers is a few tens of Pa [1, 6]. In the next section, the cell mechanical stress will be discussed depending on the viscoelasticity of multicellular systems and the cell-matrix interfacial tension.

### Cell mechanical stress generation caused by collective cell migration

The cell mechanical stress generated during collective cell migration is influenced by the viscoelasticity of epithelial monolayers and by the cell-matrix interfacial tension [38]. The viscoelasticity of epithelial monolayers and cell-matrix interfacial tension depend on the strength of cell-cell and cell-matrix adhesion contacts, and cell contractility. Both types of adhesion contacts are influenced by the stiffness of the substrate matrix.

The cell-matrix interfacial tension depends on the epithelial surface tension, matrix surface tension, and cell-matrix adhesion energy. This physical parameter is time-dependent and can be expressed as:

1 $$\:{\gamma\:}_{em}\left(r,\tau\:\right)={\gamma\:}_{e}\left(r,\tau\:\right)+{\gamma\:}_{m}\left(r,\tau\:\right)-{\omega\:}_{a}\left(r,\tau\:\right)$$

where the cell-matrix adhesion energy $\:{\omega\:}_{a}$ is released when two surfaces come into contact. The interfacial tension decreases with the strength of FAs. The equilibrium (static) tissue surface tension measured after uni-axial compression of cell aggregates is: (1) $\:4.5\:\frac{\text{mN}}{\textrm{m}}$ for F9 WT cell aggregates [39]; (2) $\:1.6\pm\:0.6\:\frac{\text{mN}}{\textrm{m}}$ to $\:4.0\pm\:1.0\:\frac{\text{mN}}{\textrm{m}}$ within 9 days for embryonic neural retina aggregates [40]; and (3) $\:22.8\pm\:3\:\frac{\text{mN}}{\textrm{m}}$ for aggregates of CHO cells [41]. The static surface tension of collagen I matrix decreases from $\:62\:\frac{\text{mN}}{\textrm{m}}$ to $\:57\:\frac{\text{mN}}{\textrm{m}}$ at $\:21\:{}^{\text{o}}\text{C}$ when the concentration of collagen increases from $\:1\:\frac{\text{mg}}{\textrm{ml}}$ to $\:4\:\frac{\text{mg}}{\textrm{ml}}$ (in experiments without cells) [42]. The inhomogeneous distribution of the strength of cell-cell and cell-matrix adhesion contacts, as well as the surface rearrangement of the substrate matrix, caused by cell tractions, lead to an inhomogeneous distribution of the interfacial tension. An inhomogeneous distribution of the epithelial surface tension causes hole formation during passive de-wetting of murine sarcoma (S-180) cell monolayers on a non-adhesive substrate matrix [21]. Pérez-González et al. [11] observed a radial distribution of E-cadherin concentration, and consequently, the epithelial surface tension within the monolayers. An inhomogeneous distribution of the matrix surface tension can be induced by rearrangement of the polymer matrix caused by cell tractions [38]. Clark et al. [26] considered the movement of A431 cell clusters on the collagen I matrix and revealed that the distribution of collagen concentration around the cell cluster is asymmetric, such that the collagen concentration near the front of the cluster is ~ 30% lower than that near its rear. The change in collagen in-plane concentration causes the establishment of a matrix surface tension gradient, which has a feedback impact on the directional migration of the cell cluster [38]. The strength of the cell FAs, as well as cell traction forces varies along the cell monolayers [43]. Strong cell-cell adhesion contacts within keratinocyte monolayers localize the traction forces to the colony periphery [43]. The main characteristic of migrating epithelial collectives is the inhomogeneous distribution of cell tractions, cell packing density, velocity, and accumulated stress [1, 30, 32]. From Eq. 1 the interfacial tension gradient can be expressed as: $\:{\overrightarrow{\nabla\:}\gamma\:}_{em}=\overrightarrow{\nabla\:}{\gamma\:}_{e}+\overrightarrow{\nabla\:}{\gamma\:}_{m}-\overrightarrow{\nabla\:}{\omega\:}_{a}$.

Consequently, both the interfacial tension and its gradient influence the generation of the cell residual stress, i.e., the stress, that remains in the cellular systems during collective cell migration and changes on a time scale of hours [7]. The cell residual stress can have both normal (tensional/compressive) and shear components. All components of the cell stress have been measured within migrating epithelial monolayers [1, 2, 6]. The cell normal residual stress includes isotropic and deviatoric parts. The isotropic part of the cell normal residual stress is induced by the work of the epithelial-matrix interfacial tension in decreasing the biointerface area expressed by the Young-Laplace equation. The deviatoric part of the cell normal stress is the viscoelastic normal stress attributed to collective cell migration. It is in accordance with fact that migrating cell groups perform directional migration which can be perturbed during inhomogeneous wetting/de-wetting. Consequently, the cumulative effects of cell-matrix interactions lead to generation of the isotropic part of the cell normal residual stress, while the deviatoric part the normal residual stress is generated internally within multicellular systems. Consequently, the cell normal residual stress can be expressed as:

2 $$\:{\stackrel{\sim}{\varvec{\sigma\:}}}_{\varvec{c}\varvec{r}\varvec{V}}=\pm\:\varDelta\:{p}_{c\to\:m}\stackrel{\sim}{\varvec{I}}+{{\stackrel{\sim}{\varvec{\sigma\:}}}_{\varvec{c}\varvec{r}\varvec{V}}}^{\varvec{C}\varvec{C}\varvec{M}\:}$$

where $\:{\stackrel{\sim}{\varvec{\sigma\:}}}_{\varvec{c}\varvec{r}\varvec{V}}$ is the cell normal residual stress part, which includes $\:{\sigma\:}_{crxx}$ and $\:{\sigma\:}_{cryy}$ components, $\:\stackrel{\sim}{\varvec{I}}$ is the unity tensor, $\:\varDelta\:{p}_{c\to\:m}$ is the isotropic part of the cell normal stress equal to $\:\varDelta\:{p}_{c\to\:m}=-{\gamma\:}_{em}\left(\overrightarrow{\nabla\:}\cdot\:\overrightarrow{\varvec{n}}\right)$, $\:\overrightarrow{\varvec{n}}$ is the normal vector of the cell-matrix biointerface, and $\:{{\stackrel{\sim}{\varvec{\sigma\:}}}_{\varvec{c}\varvec{r}\varvec{V}}}^{\varvec{C}\varvec{C}\varvec{M}\:}$ is the deviatoric part of the cell normal residual stress with the components $\:{{\sigma\:}_{crxx}}^{CCM}$ and $\:{{\sigma\:}_{cryy}}^{CCM}$. The positive and negative signs of the isotropic stress part indicate tension and compression, respectively. The deviatoric part of normal stress depends on the viscoelasticity of epithelial monolayers. While passive wetting/de-wetting generates an isotropic contribution to the cell normal residual stress (caused by effects along the epithelial-matrix biointerface), collective cell migration during active wetting/de-wetting generates an anisotropic (i.e., deviatoric) contribution to the normal residual stress. The viscoelasticity further depends on the cell packing density and the strength of the cell-cell adhesion contacts, which will be discussed in more detail.

The inhomogeneous distribution of the cell normal stress components, generated during collective cell migration, causes an inhomogeneous distribution of cell packing density within monolayers. Three subpopulations can be distinguished:

1. A migratory, proliferative subpopulation with the cell packing density $\:{n}_{e}<{n}_{h}$ (where $\:{n}_{h}$ is the cell packing density at homeostasis and $\:{n}_{e}$ is the epithelial packing density);
2. A homeostatic cell subpopulation with cell packing density $\:{n}_{e}\sim{n}_{h}$, which satisfies the condition that proliferation is inhibited; and
3. A jamming cell subpopulation with cell packing density $\:{n}_{e}\sim{n}_{j}$, (where $\:{n}_{j}$ is the cell packing density at the jamming state), which satisfies the condition that proliferation and locomotion are inhibited.

**Fig. 1:**
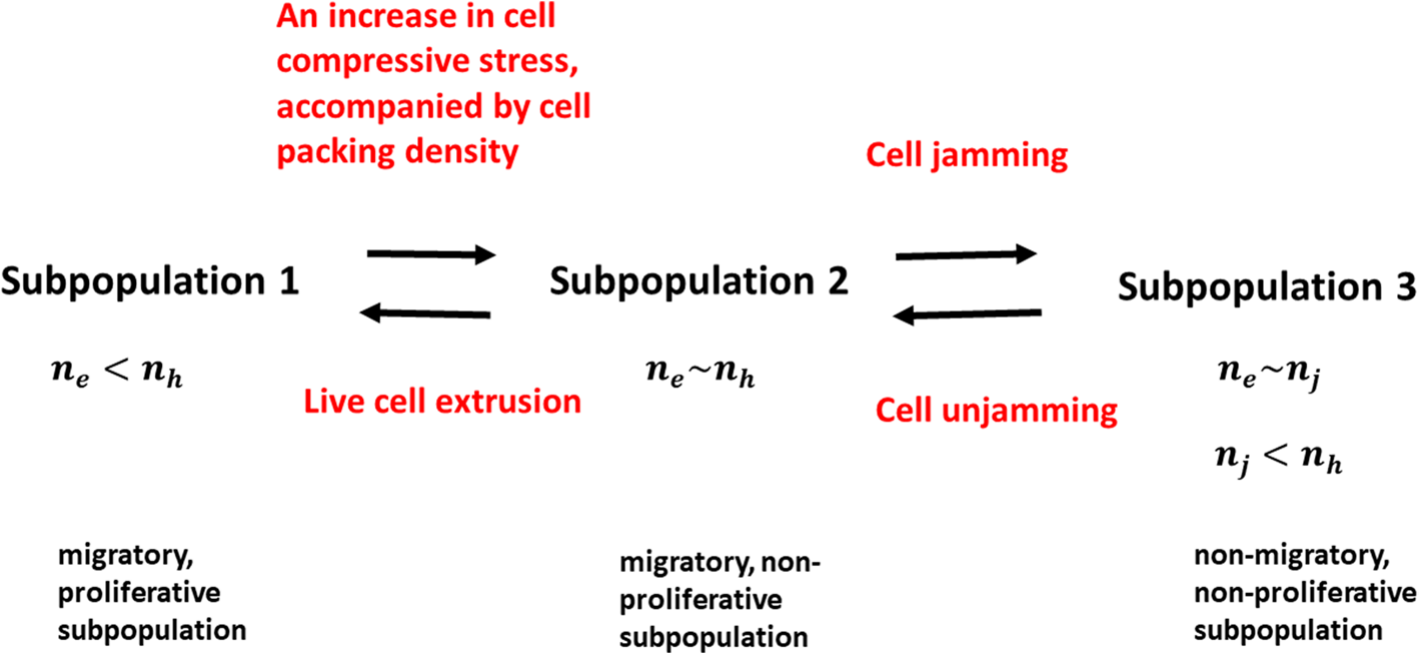
Schematic presentation of the interrelation between the three types of subpopulations within migrating epithelial monolayers

The existence of subpopulations 2 and 3 is related primarily to the accumulation of cell compressive stress [44]. The cell packing density of the jamming subpopulation is lower than that of the migratory and homeostatic subpopulations, i.e. $\:{n}_{j}<{n}_{h}$ [44]. This phenomenon, observed by Kaliman et al. [44], has not yet been explained. We will offer an explanation from the standpoint of physics in the next section. The cell packing densities, characteristic for subpopulations 2 and 3, depend on the cell type and matrix stiffness [44]. The dynamical interrelation between subpopulations is presented schematically in Fig. 1:

An increase in compressive stress drives the forwards transition from subpopulation 1 to subpopulation 2, while cell extrusion induces the transition backwards from subpopulation 2 to subpopulation 1. The transition from subpopulation 2 is also possible to subpopulation 3 and vice versa during cell jamming/unjamming. A special interest here is to understand the main properties of the cell-cell interactions, which lead to the transition from subpopulation 2 to subpopulations 1 and 3.

Tlili et al. [30] considered the active wetting of MDCK epithelial monolayers and revealed that cell packing density varies from $\:{1 \times 10}^{5}\:\frac{\text{cells}}{{\textrm{cm}}^{2}}$ to $\:{5 \times 10}^{5}\:\frac{\text{cells}}{{\textrm{cm}}^{2}}$. An increase in cell packing density from $\:{1 \times 10}^{5}\:\frac{\text{cells}}{{\textrm{cm}}^{2}}$ to $\:{5 \times 10}^{5}\:\frac{\text{cells}}{{\textrm{cm}}^{2}}$ resulted in a decrease in cell velocity from $\:0.8\:\frac{{\upmu\:}\text{m}}{\textrm{min}}$ to zero [30]. Nnetu et al. [32] pointed out that the velocity of epithelial MCF-10 A cells drops to zero at a cell packing density of $\:\sim{3.5 \times 10}^{5}\:\frac{\text{cells}}{{\textrm{cm}}^{2}}$, corresponding to the cell jamming. Petitjean et al. [45] revealed that the MDCK cell monolayers reached the confluence for a cell packing density of $\:{n}_{conf}\sim2.5x{10}^{5}\:\frac{\text{cells}}{{\textrm{cm}}^{2}}$ and a cell velocity of $\:\sim0.14\:\frac{{\upmu\:}\text{m}}{\textrm{min}}$.

External compression of confluent MDCK cell monolayers with 28% strain caused an increase in cell packing density to $\:1.39x{n}_{conf}$, which stimulated the extrusion of live cells [4]. In this case, the corresponding fraction of extruded cells reached 6% [4]. Consequently, the cell packing density under jamming is higher than or equal to the cell packing density for the cell extrusion. A detailed description of the underlying physical mechanisms will be discussed in the next two sections.

The cell shear residual stress includes two parts. One part is generated by natural convection as a consequence of the existence of the interfacial tension gradient $\:\overrightarrow{\nabla\:}{\gamma\:}_{em}$, while the other part is generated by forced convection (i.e., by collective cell migration). The cell active/passive extension from the multicellular domains of lower interfacial tension towards the domains of higher interfacial tension is part of the Marangoni effect [46]. The phenomenon of cell movement along multicellular surfaces caused by the surface tension gradient has been confirmed experimentally by Gsell et al. [47]. The Marangoni effect has also been recognized in various soft matter systems under temperature or concentration gradients [48].

Consequently, the cell shear residual stress can be expressed as:

3 $$\:\overrightarrow{\varvec{n}}\cdot\:{\stackrel{\sim}{\varvec{\sigma\:}}}_{\varvec{e}\varvec{r}\varvec{S}}\cdot\:\overrightarrow{\varvec{t}}=\overrightarrow{\nabla\:}{\gamma\:}_{em}\cdot\:\overrightarrow{\varvec{t}}+\overrightarrow{\varvec{n}}\cdot\:{{\stackrel{\sim}{\varvec{\sigma\:}}}_{\varvec{e}\varvec{r}\varvec{S}}}^{CCM\:}\cdot\:\overrightarrow{\varvec{t}}$$

where $\:{\stackrel{\sim}{\varvec{\sigma\:}}}_{\varvec{e}\varvec{r}\varvec{S}}$ is the cell shear residual stress component, which is symmetric and satisfies the condition that the corresponding components are $\:{\sigma\:}_{crxy}={\sigma\:}_{cryx}$, $\:{{\stackrel{\sim}{\varvec{\sigma\:}}}_{\varvec{e}\varvec{r}\varvec{S}}}^{CCM\:}$ is the cell shear residual stress generated by collective cell migration with the component $\:{{\sigma\:}_{crxy}}^{CCM}$, and $\:\overrightarrow{\varvec{t}}$ is the tangent vector of the cell-matrix biointerface. The gradient of interfacial tension can be expressed as $\:\frac{\varDelta\:{\gamma\:}_{em}}{\varDelta\:L}$ (where $\:\varDelta\:{\gamma\:}_{em}$ is the interfacial tension difference and $\:\varDelta\:L$ is the distance in which this gradient exist). If it is supposed that the interfacial tension difference corresponds to only $\:\varDelta\:{\gamma\:}_{em}\approx\:1\:\frac{\text{mN}}{\textrm{m}}$ and the distance is $\:\varDelta\:L\approx\:100\:{\upmu\:}\text{m}$ (i.e., an order of magnitude larger than the size of single cell), this gradient of the interfacial tension corresponds to a cell shear stress part of $\:\sim10\:\text{Pa}$. This is a relatively large value, bearing in mind that a cell shear stress of a few tens of Pa can induce inflammation of epithelial cells [49].

The cell shear/normal residual stress caused by collective cell migration depends on the mechanism of cell migration, and on that basis, it depends on the cell packing density. Epithelial cell migration occurs via: (1) the convective mechanism for the cell packing density $\:{n}_{e}\le\:{n}_{conf}$, (2) the diffusion mechanism for the cell packing density $\:{{n}_{conf}<n}_{e}<{n}_{h}$, and (3) the sub-diffusion mechanism for the cell packing density $\:{n}_{e}\sim{n}_{h}$ [3, 50]. Constitutive models proposed for various modes of epithelial cell migration are shown in Table 1.

**Table 1:** Some constitutive models proposed for various modes of epithelial cell migration

Modes of epithelial cell migration	Constitutive models for viscoelasticity of epithelial tissues
Convective cell migration mode $\:{n}_{e}\le\:{n}_{conf}$ $\:0.1\:<||{\overrightarrow{\varvec{v}}}_{\varvec{e}}||<\sim1\:\frac{\mu\:m}{min}$	The Zener model for viscoelastic solids: $\:{{\stackrel{\sim}{\varvec{\sigma\:}}}_{\varvec{e}\varvec{k}}}^{CCM}\left(r,t,\tau\:\right)+{\tau\:}_{Rck}\:{{\dot{\stackrel{\sim}{\varvec{\sigma\:}}}}_{\varvec{e}\varvec{k}}}^{CCM}={E}_{ck}{\stackrel{\sim}{\varvec{\epsilon\:}}}_{\varvec{e}\varvec{k}}\left(r,\tau\:\right)+{\eta\:}_{ck}{\dot{\stackrel{\sim}{\varvec{\epsilon\:}}}}_{\varvec{e}\varvec{k}}\:$ Stress relaxation under constant strain condition $\:{\stackrel{\sim}{\varvec{\epsilon\:}}}_{0\varvec{c}\varvec{k}}$ per single short-time relaxation cycle: $\:{{\stackrel{\sim}{\varvec{\sigma\:}}}_{\varvec{e}\varvec{k}}}^{CCM}\left(r,t,\tau\:\right)={\stackrel{\sim}{\varvec{\sigma\:}}}_{0\varvec{e}\varvec{k}}{e}^{-\frac{t}{{\tau\:}_{Rck}}}+{{\stackrel{\sim}{\varvec{\sigma\:}}}_{\varvec{r}\varvec{e}\varvec{k}}}^{CCM}\left(r,\tau\:\right)\left(1-{e}^{-\frac{t}{{\tau\:}_{Rck}}}\right)$ Cell residual stress is elastic. $\:{{\stackrel{\sim}{\varvec{\sigma\:}}}_{\varvec{r}\varvec{e}\varvec{k}}}^{CCM}={E}_{ck}\:{\stackrel{\sim}{\varvec{\epsilon\:}}}_{\varvec{e}\varvec{k}}$
Diffusion cell migration mode $\:{n}_{h}>{n}_{e}>{n}_{conf}$ $\:||{\overrightarrow{\varvec{v}}}_{\varvec{e}}||\sim{10}^{-3}-{10}^{-2}\frac{\mu\:m}{min}$	The Kelvin-Voigt model for viscoelastic solids: $\:{{\stackrel{\sim}{\varvec{\sigma\:}}}_{\varvec{e}\varvec{k}}}^{CCM}\left(r,\tau\:\right)={E}_{ck}{\stackrel{\sim}{\varvec{\epsilon\:}}}_{\varvec{e}\varvec{k}}+{{\eta\:}_{ck}\:\dot{\stackrel{\sim}{\varvec{\epsilon\:}}}}_{\varvec{e}\varvec{k}}$ The stress cannot relax. $\:{{\stackrel{\sim}{\varvec{\sigma\:}}}_{\varvec{e}\varvec{k}}}^{CCM}={{\stackrel{\sim}{\varvec{\sigma\:}}}_{\varvec{r}\varvec{e}\varvec{k}}}^{CCM}$
Sub-diffusion cell migration mode $\:{n}_{e}\sim{n}_{h}$ $\:||{\overrightarrow{\varvec{v}}}_{\varvec{e}}||\to\:0$	The fraction model for viscoelastic solids: $\:{{\stackrel{\sim}{\varvec{\sigma\:}}}_{\varvec{e}\varvec{k}}}^{CCM}\left(r,\tau\:\right)={{\upeta\:}}_{\alpha\:k}{D}^{\alpha\:}\left({\stackrel{\sim}{\varvec{\epsilon\:}}}_{\varvec{e}\varvec{k}}\right)$ For $\:0<\alpha\:<1/2$ The stress cannot relax. $\:{{\stackrel{\sim}{\varvec{\sigma\:}}}_{\varvec{e}\varvec{k}}}^{CCM}={{\stackrel{\sim}{\varvec{\sigma\:}}}_{\varvec{r}\varvec{e}\varvec{k}}}^{CCM}$

where the subscript $\:k\equiv\:S,V$, $\:S$ is shear, $\:V$ is volumetric, $\:{\tau\:}_{Rck}$ is the cell stress relaxation time, $\:{E}_{ck}$ is the elastic modulus, $\:{{\upeta\:}}_{ck}$ is the cell viscosity (shear or bulk), $\:r$ is the space coordinate, $\:t$ is a short-time scale (i.e. minutes), $\:\tau\:$ is a long-time-scale (i.e. hours), $\:||{\overrightarrow{\varvec{v}}}_{\varvec{e}}||$ is the cell speed, $\:{\overrightarrow{\varvec{v}}}_{\varvec{e}}$ is the cell velocity equal to $\:{\overrightarrow{\varvec{v}}}_{\varvec{e}}=\frac{\varvec{d}\overrightarrow{\varvec{u}}}{\varvec{d}\varvec{\tau\:}}$, $\:\overrightarrow{\varvec{u}}\left(r,\tau\:\right)$ is the cell local displacement field, $\:{{\stackrel{\sim}{\varvec{\sigma\:}}}_{\varvec{e}\varvec{k}}}^{CCM}\left(r,t,\tau\:\right)$ is the cell stress (normal or shear), $\:{{\dot{\stackrel{\sim}{\varvec{\sigma\:}}}}_{\varvec{e}\varvec{k}}}^{CCM}$ is the rate of stress change $\:{{\dot{\stackrel{\sim}{\varvec{\sigma\:}}}}_{\varvec{e}\varvec{k}}}^{CCM}=\frac{d{{\stackrel{\sim}{\varvec{\sigma\:}}}_{\varvec{e}\varvec{k}}}^{CCM}}{dt}$ caused by the stress relaxation, $\:{\stackrel{\sim}{\varvec{\epsilon\:}}}_{\varvec{c}\varvec{k}}$ is the cell strain such that the volumetric strain is equal to $\:{\stackrel{\sim}{\varvec{\epsilon\:}}}_{\varvec{e}\varvec{V}}\left(r,\tau\:\right)=\overrightarrow{(\nabla\:}\cdot\:\overrightarrow{\varvec{u}})\stackrel{\sim}{\varvec{I}}$, $\:\stackrel{\sim}{\varvec{I}}$ is the unit tensor, and the shear strain $\:{\stackrel{\sim}{\varvec{\epsilon\:}}}_{\varvec{e}\varvec{S}}\left(r,\tau\:\right)=\frac{1}{2}\left(\overrightarrow{\nabla\:}\overrightarrow{\varvec{u}}+{\overrightarrow{\nabla\:}\overrightarrow{\varvec{u}}}^{\varvec{T}}\right)$, $\:{\dot{\stackrel{\sim}{\varvec{\epsilon\:}}}}_{\varvec{e}\varvec{k}}$ is the corresponding strain rate equal to $\:{\dot{\stackrel{\sim}{\varvec{\epsilon\:}}}}_{\varvec{e}\varvec{k}}=\frac{d{\stackrel{\sim}{\varvec{\epsilon\:}}}_{\varvec{c}\varvec{k}}}{d\tau\:}$, $\:{{\upeta\:}}_{\alpha\:k}$ is the effective modulus, $\:{D}^{\alpha\:}\:\stackrel{\sim}{\varvec{\epsilon\:}}\left(r,\tau\:\right)=\frac{{d}^{\alpha\:}\stackrel{\sim}{\varvec{\epsilon\:}}\left(r,\tau\:\right)}{d{\tau\:}^{\alpha\:}}$ is the fractional derivative, and $\:{\upalpha\:}$ gives the order of fractional derivatives (the damping coefficient). Caputo’s definition of the fractional derivative of a function $\:\stackrel{\sim}{\varvec{\epsilon\:}}\left(r,\tau\:\right)$ is used and expressed as: $\:{D}^{\alpha\:}\stackrel{\sim}{\varvec{\epsilon\:}}=\frac{1}{\lceil\left(1-\alpha\:\right)}\frac{d}{dt}{\int\:}_{0}^{t}\frac{\stackrel{\sim}{\varvec{\epsilon\:}}\left({r,\tau\:}^{{\prime\:}}\right)}{{\left(\tau\:-\tau\:{\prime\:}\right)}^{\alpha\:}}d\tau\:{\prime\:}$ (where Г$\:\left(1-\alpha\:\right)$ is a gamma function) [51]

Serra-Picamal et al. [1] and Notbohm et al. [2] considered the rearrangement of MDCK cell monolayers with the cell packing density $\:{n}_{e}\le\:{n}_{conf}$ and revealed that the long-term cell stress (i.e., the cell residual stress) correlates with the corresponding strain, pointing out the viscoelastic solid behaviour. It is in accordance with the fact that epithelial cells establish strong E-cadherin mediated cell-cell adhesion contacts. Another important behaviour of epithelial monolayers, characteristic for this regime of cell packing density, is the ability of cell stress to relax towards the cell residual stress. Khalilgharibi et al. [52] reported that the stress relaxation time corresponds to a time-scale of minutes, while the cell residual stress accumulation occurs on a time-scale of hours [7]. The stress relaxation ability caused by uni-axial compression of cell aggregates was observed by Marmottant et al. [53]. Based on these findings, Pajic-Lijakovic and Milivojevic [7] concluded that cell stress change occurs through many short-time stress relaxation cycles, while cell strain (induced by cell movement) and corresponding cell residual stress change over a time scale of hours. A suitable constitutive model, satisfying the conditions (1) that the stress relaxes exponentially on a time scale of minutes and (2) that the cell residual stress correlates with the corresponding strain, pointing to long-term elastic behaviour, could be the Zener model presented in Table 1 [18]. In this case, energy dissipation, characteristic of the viscoelastic behaviour of multicellular systems, occurs on a time scale of minutes as a consequence of the remodelling of cell-cell adhesion contacts [3]. The cell stress relaxes towards the elastic cell residual stress. Cell residual stress, cell velocity and corresponding strain, oscillate on a time scale of hours, which has been discussed in the context of mechanical waves [1, 2, 7]. In this case, the cell actual stress can be expressed as: $\:{\stackrel{\sim}{\varvec{\sigma\:}}}_{\varvec{e}\varvec{i}}\left(r,t,\tau\:\right)={\stackrel{\sim}{\varvec{\sigma\:}}}_{\varvec{e}\varvec{r}\varvec{i}}\left(r,\tau\:\right)+\varDelta\:{{\stackrel{\sim}{\varvec{\sigma\:}}}_{\varvec{e}\varvec{i}}}^{\varvec{C}\varvec{C}\varvec{M}\:}\left(r,t,\tau\:\right)$ (where $\:i\equiv\:V,S$ is the subscript in Eqs. 2 and 3, which indicates normal and shear stress components, $\:{\stackrel{\sim}{\varvec{\sigma\:}}}_{\varvec{e}\varvec{r}\varvec{i}}\left(r,\tau\:\right)$ is the cell residual stress (normal and shear) expressed by Eqs. 2 and 3, $\:\varDelta\:{{\stackrel{\sim}{\varvec{\sigma\:}}}_{\varvec{e}\varvec{i}}}^{\varvec{C}\varvec{C}\varvec{M}\:}$ is an increment of the actual cell stress change during a single short-time stress relaxation cycle equal to $\:\varDelta\:{{\stackrel{\sim}{\varvec{\sigma\:}}}_{\varvec{e}\varvec{i}}}^{\varvec{C}\varvec{C}\varvec{M}\:}\left(r,t,\tau\:\right)={{\stackrel{\sim}{\varvec{\sigma\:}}}_{\varvec{e}\varvec{i}}}^{\varvec{C}\varvec{C}\varvec{M}\:}\left(r,t,\tau\:\right)-{\stackrel{\sim}{\varvec{\sigma\:}}}_{\varvec{e}\varvec{r}\varvec{i}}\left(r,\tau\:\right)$ and $\:{{\stackrel{\sim}{\varvec{\sigma\:}}}_{\varvec{e}\varvec{i}}}^{\varvec{C}\varvec{C}\varvec{M}\:}\left(r,t,\tau\:\right)$ is the part of actual stress caused by collective cell migration, which is expressed by the Zener model and presented in Table 1 for the cell packing density $\:{n}_{e}\le\:{n}_{conf}$).

Further increase in the cell packing density, in the range of $\:{{n}_{conf}<n}_{e}<{n}_{h}$, results in suppression of the cell stress relaxation. Cell-cell frictional effects, characteristic of higher cell packing densities, lead to a long term dissipation of energy during cell rearrangement. In accordance with the fact that a linear, diffusion mechanism underlies cell movement, the corresponding constitutive model should also be linear. Pajic-Lijakovic and Milivojevic [18] proposed the Kelvin-Voigt constitutive model for this regime (Table 1). Corresponding long-term changes in cell stress account for both the elastic and viscous contributions. In this case, cell actual stress is equal to cell residual stress.

While the viscoelasticity of epithelial monolayers shows linear behaviour for cell packing densities $\:{n}_{e}<{n}_{h}$, the damped cell movement, described by the sub-diffusion mechanism, induces nonlinearity in the viscoelastic behaviour. For describing the damped movement of cells at homeostasis, it is necessary to use fractional derivatives. Pajic-Lijakovic and Milivojevic [50] proposed the fractional stress-strain model for this regime of viscoelasticity (Table 1). In this case too, cell actual stress is equal to cell residual stress.

Cell actomyosin contractility has two main effects on cellular behaviour. First, it enhances the strength of E-cadherin adhesion contacts, which in turn affects the surface tension of the epithelial monolayer. Secondly, it induces cell tractions, which then influence the surface tension of the extracellular matrix and the energy of epithelial-matrix adhesion. As a result, cell contractility plays a crucial role in determining the interfacial tension between the epithelial monolayer and the matrix, as well as the gradient of this tension. This, in turn, affects the cell mechanical stress. The impact of cell contractility on the strength of cell-cell adhesion contacts also has implications for the viscoelasticity of the epithelial monolayer. Active (contractile) cells exhibit greater stiffness compared to non-contractile cells, primarily due to the accumulation of contractile energy. Research by Schulze et al. [54] has shown that the Young’s modulus of contractile MDCK cell monolayers is approximately 33.0 ± 3.0 kPa, while non-contractile cells have a modulus that is roughly half of this value. Furthermore, active wetting and de-wetting processes lead to the generation of higher cell residual stress compared to passive wetting and de-wetting under the same strain conditions. It is important to note that cell contractility affects all physical parameters involved in the generation of cell mechanical stress, making it impossible to separate the stress into active and passive contributions.

In summary, cell actomyosin contractility plays a significant role in modulating the behaviour of epithelial cells. Its effects on adhesion contacts, surface tension, and mechanical stress have important implications for the overall mechanical properties and behaviour of epithelial monolayers. Cell mechanical stress, generated during cell active wetting/de-wetting, can induce the formation of the topological defects in cell alignment which, has a feedback impact on cell rearrangement.

## The generation of topological defects in cell alignment occurred in an overcrowded environment: cell-cell interactions

**Fig. 2:**
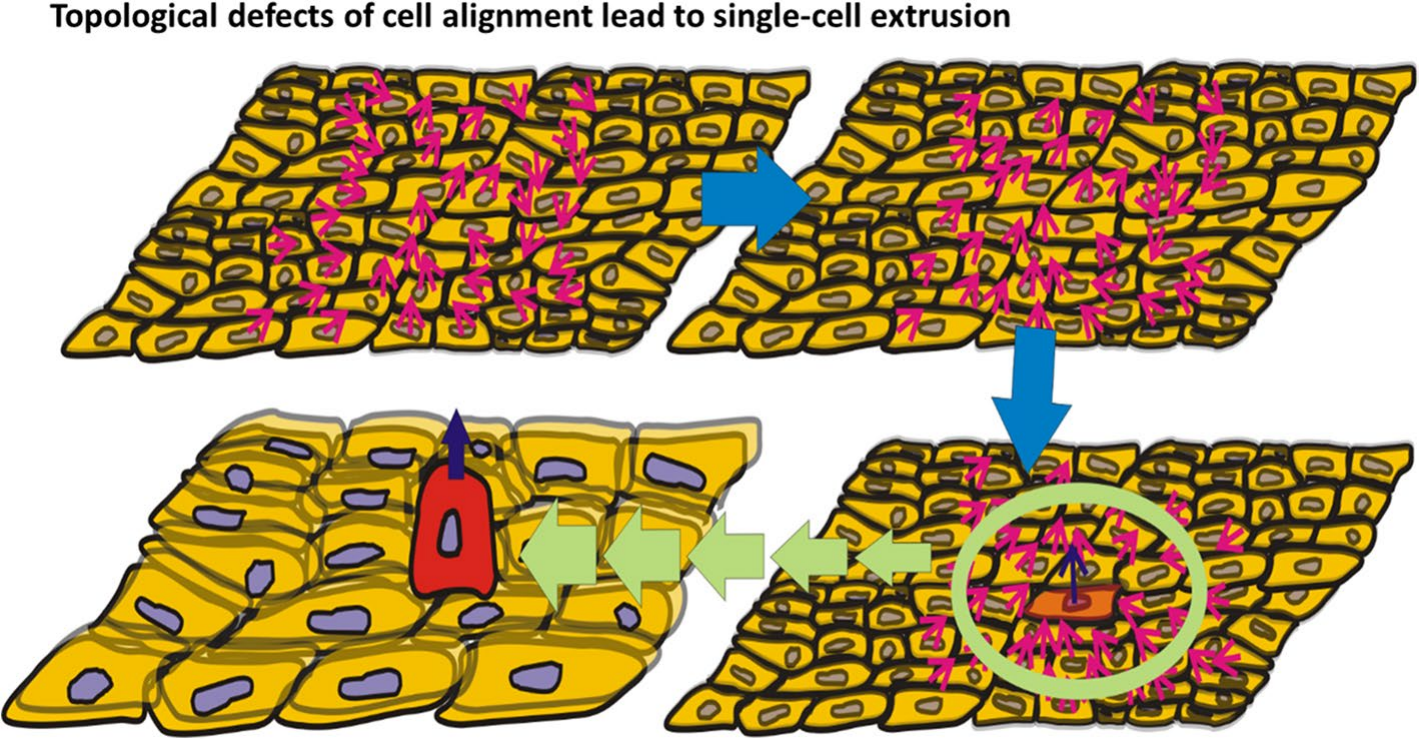
A topological defect in cell alignment occur in an overcrowded environment, leads to single-cell extrusion. These defects are induced by the interplay between cell compressive and shear stress components

Topological defects arise as a perturbation of cell flow-polarity alignment caused by cell-cell interactions within an overcrowded environment [5] as was shown in Fig. 2:

Saw et al. [5] identified the isotropic part of the cell compressive stress, while Ohsawa et al. [55] proposed the cell crowding stress, as the main physical factor responsible for the generation of defects. However, the cell crowding stress was not clearly connected with the isotropic part of the cell compressive stress. We emphasize that the isotropic and anisotropic (i.e., deviatoric) parts of cell compressive stress both contribute to the generation of topological defects. An increase of the compressive stress causes an increase in cell packing density leading to the formation of overcrowded regions. Besides the cell compressive stress, it would be necessary to include the cell shear stress in the formation of the topological defects. While compressive stress stimulates cell-cell interactions, the gradient of interfacial tension (as a part of the cell shear stress) can perturb cell alignment by inducing passive cell movement from the region of lower, to higher, cell-matrix interfacial tension [38].

### Cell-cell interactions in an overcrowded environment

Cell-cell mechanical interactions in overcrowded regions trigger various signalling cascades to prevent cell overlap and reduce collisions (i.e., a decrease in cell-cell distance). The decrease in collisions between cells can be achieved by either inhibiting cell movement under constant cell packing density or by reducing the density of packed cells while maintaining their ability to migrate. Contact inhibition of locomotion, resulting from head-on interactions, can restrict cell movement [15–17]. On the other hand, live cell extrusion, caused by cell glancing interactions, contributes to a reduction in cell packing density [4]. A more detailed description of induced cell signalling caused by cell interactions will be provided in Sect. 5.1 and 5.2. In this line, two types of cell-cell interactions will be considered: (1) cell head-on interactions and (2) cell glancing interactions.

**Fig. 3:**
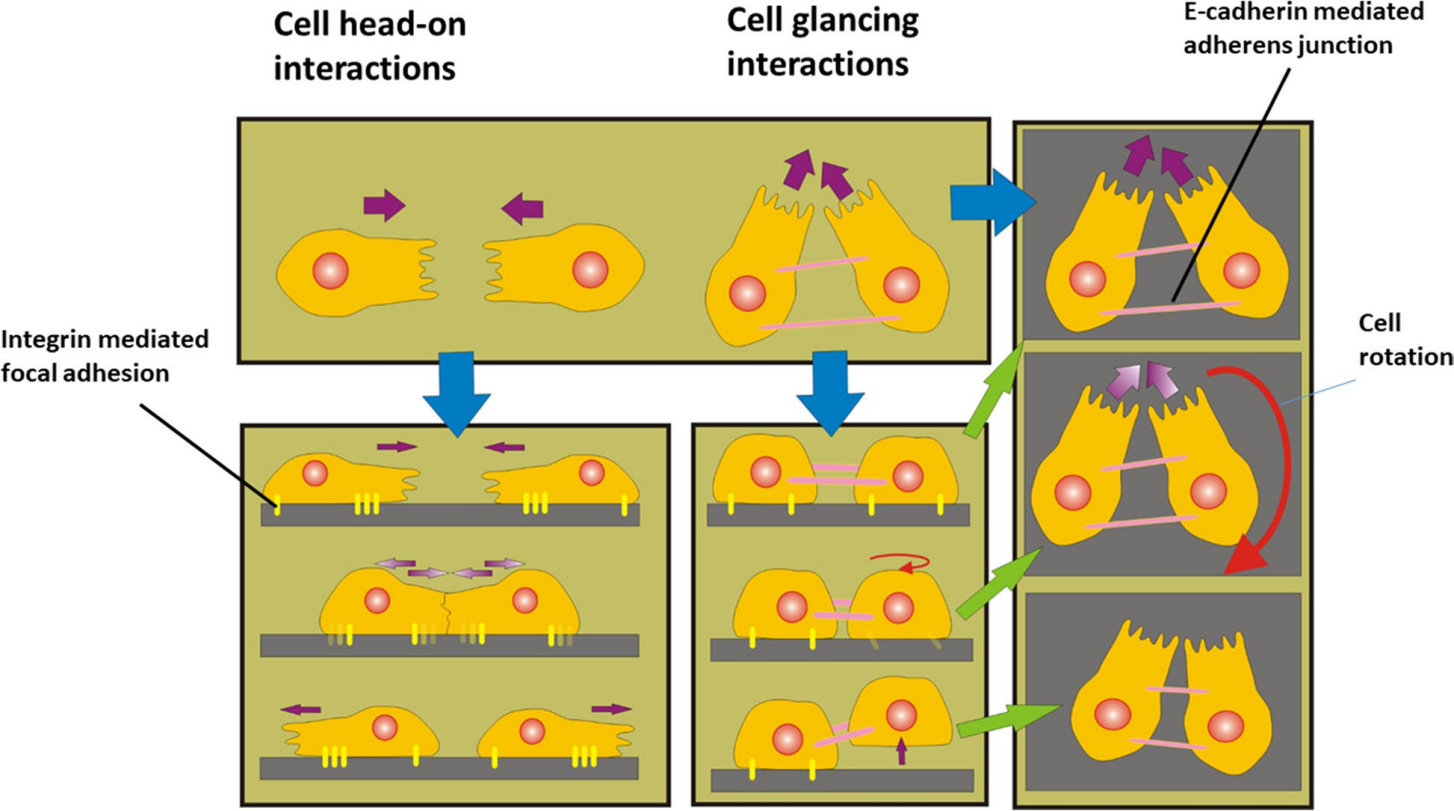
Cell-cell interactions: head-on interactions and glancing interactions. Purple arrows represent the direction of cell movement; the red arrow represents a single-cell rotation. Cell glancing interactions, caused by perturbation of cell alignment, can induce live cell extrusion. Cell head-on interactions have been discussed in the context of cell jamming

The head-on interactions induce cell re-polarisation and weakening of cell-cell and cell-matrix adhesion contacts, resulting in a change in the direction of cell movement [16]. While cell head-on interactions have been widely studied in the context of cell contact inhibition of locomotion [15, 16], the role of cell glancing interactions in cell rearrangement has started to be elucidated. Glancing interactions are a form of cell orientational interaction that occurs as cells align themselves in the direction of collective cell migration. Cadherin-mediated cell-cell adhesion contacts play a crucial role in this alignment process and directional cell migration [56]. Weakening of cell-cell adhesion contacts results in a decrease in the size of the region of topological defects of cell alignment [5]. Perturbation of cell alignment leads to the imbalance of intercellular forces responsible for cell realignment causing the re-establishment of the force balance [20]. In accordance with fact that intercellular force acts through cell-cell adhesion contacts, this force imbalance can be connected with an inhomogeneous distribution of the strength of adherens junctions [57]. In this line, it is evident that some cell-cell adhesion contacts are more stretched, and stronger, while others are less stretched or even compressed, and weaker. Guevorkian et al. [27] demonstrated experimentally that stretching of epithelial cells leads to an increase in the strength of cell-cell adhesion contacts and consequently, an increase in epithelial surface tension. The inhomogeneous distribution of intercellular forces generates a torque $\:\varDelta\:\overrightarrow{\varvec{T}}$. This torque, responsible for cell re-alignment, is $\:\varDelta\:\overrightarrow{\varvec{T}}={\overrightarrow{\varvec{F}}}_{\varvec{c}}X{\overrightarrow{\varvec{r}}}_{\varvec{c}}$ where $\:{\overrightarrow{F}}_{c}$ is the resultant force generated by the extension/compression of the E-cadherin bonds per single cell $\:{\overrightarrow{F}}_{c}=\sum_{i=1}^{{N}_{b}}{\overrightarrow{F}}_{i}$, $\:{N}_{b}$ is the number of bonds per single adherens junction AJ which depends on the stretching/compression of AJ, $\:{\overrightarrow{F}}_{i}$ is the force per single bond, and $\:{\overrightarrow{\varvec{r}}}_{\varvec{c}}$ is the radius of cell rotation, which is approximately equal to the cell radius. The number of established E- cadherin bonds between neighbouring cells and expressed per single cell is in the range of $\:10-{10}^{3}\frac{\text{bonds}}{{{\upmu\:}\textrm{m}}^{2}}$ [39]. The intra-cellular tugging force is in the range $\:20\:\text{nN}\leq{\overrightarrow{\boldsymbol{F}}}_{\boldsymbol c}\leq\:100\:\text{nN}$, while the required force for breakage of a single E-cadherin bond is ~ 200 nN [58]. The size of single adherens junction AJ is $\:{20\:{{\upmu\:}\text{m}}^{2}\le\:A}_{c}\le\:100\:{{\upmu\:}\text{m}}^{2}$ [58]. The work done per unit time by the torque $\:\varDelta\:\overrightarrow{\varvec{T}}$, which induces single-cell rotation in order to re-align the cell, is $\:W=\varDelta\:\overrightarrow{\varvec{T}}\cdot\:{\overrightarrow{\varvec{\omega\:}}}_{\varvec{c}}$ where $\overrightarrow{{\mathrm\omega}_{\mathrm c}}=\frac{d\overrightarrow{\boldsymbol{\theta}}}{d\tau}$ is the angular velocity and $\overrightarrow{\boldsymbol{\theta}}$ is the angle of cell rotation. Moreover, cell glancing interactions can induce inhomogeneity of the epithelial surface tension and cell-matrix interfacial tension on a cellular level. The epithelial surface tension gradient and the interfacial tension gradient can be correlated with the crowding stress proposed by Ohsawa et al. [55]. Both types of interactions are shown in Fig. 3.

This cell rotation generates torsional stress on the cell-matrix focal adhesions (FAs) by stimulating the detachment of FAs. The torsional shear strain can be expressed as $\:{\epsilon\:}_{mt}=\frac{{r}_{t}}{L}{\theta\:}_{t}$ where $\:{r}_{t}$ is the radius of the FA domain, $\:L$ is the thickness of the FA, and $\:{\theta\:}_{t}$ is the torsion angle. FAs are flat, elongated structures 1–5 μm long, 300–500 nm wide and, on average, 50 nm thick [59]. Consequently the corresponding torsional shear strain on an FA, calculated for $\:{r}_{t}=2\:{\upmu\:}\text{m}$, $\:L=50\:\text{nm}$, and the angle of $\:{\theta\:}_{t}={0.5}^{o}$ ($\:0.00872\:\text{rad}$) is $\:{\epsilon\:}_{mt}=0.35$. A shear strain of $\:\sim0.2$ applied on 1 mg/ml collagen I matrix without cells causes an increase in the shear stress within the matrix to 18 Pa and then relaxes towards the matrix residual stress of 4 Pa within 5 min [60]. Paddillaya et al. [61] revealed that the cell-matrix interfacial shear stress of 4–6 Pa is enough to cause the detachment of an FA. These cell-cell interactions trigger cells to activate mechanisms for regulation of the compressive stress accompanying increased cell packing density.

The main characteristics of both types of cell-cell interactions are summarised in Table 2:

**Table 2:** The main characteristics of cell head-on and glancing interactions

Type of cell-cell interactions	Head-on interactions	Glancing interactions
Cell re-polarisation	yes	no
Weakening of cell-cell adhesion contacts	yes	no
Weakening of cell-matrix adhesion contacts (i.e., focal adhesions)	yes	yes
Generation of cell torque	no	yes

Cell head-on interactions are more efficient than cell glancing interactions at inducing cell re-polarisation [16]. Re-polarisation is a complex process in which a cell exchanges its front-rear polarity. This cell reorganisation leads to weakening of cell-cell and cell matrix adhesion contacts. Intensive cell-cell interactions in an overcrowded environment can extend the time needed for cell re-polarisation or even block it [18]. Notbohm et al. [2] pointed out that the average repolarization time during the rearrangement of confluent MDCK cell monolayers is $\:1.28\:\text{h}$. In contrast to cell head-on interactions, cell glancing interactions cannot induce cell re-polarisation leading weakening of cell-cell adhesion contacts. The weakening of FAs, in this case, can be induced mechanically by the generation of cell torque.

## Cell response under compressive stress: the cell jamming or live cell extrusion

**Fig. 4:**
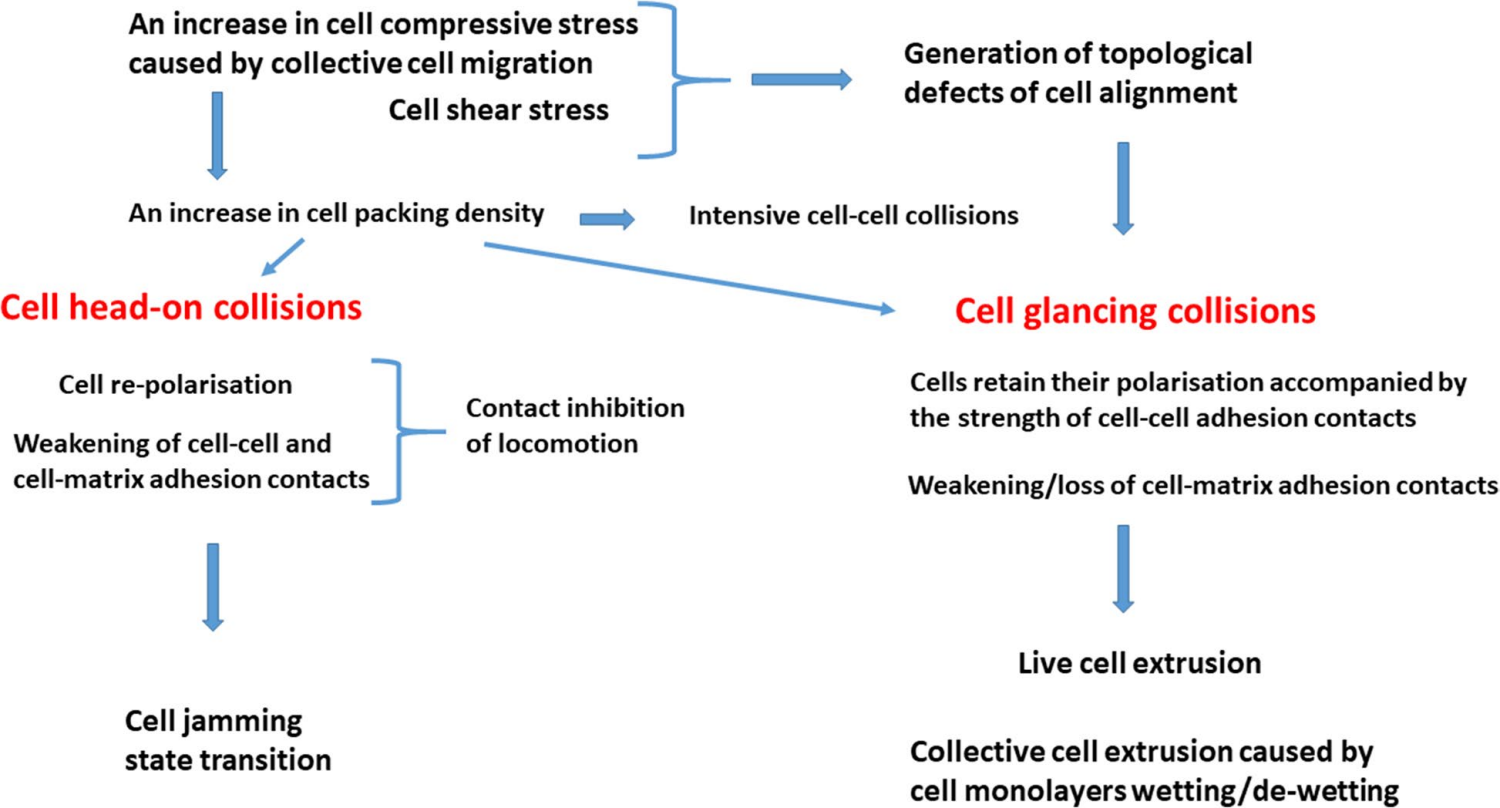
The interrelationships between various types of cell-cell interactions and cell processes such as cell jamming and live cell extrusion

The mechanisms of cell response under compressive stress are connected with the interplay between various cellular processes, such as: (1) cell signalling associated with stretch-activated ion channels, (2) remodelling of cell-cell and cell-matrix adhesion contacts, (3) change in cell contractility, and (4) a resultant decrease in the cell packing density. The physical mechanism of the compressive stress reduction will be discussed in the context of cell jamming/unjamming transitions and live cell extrusion. The interrelationships between various types of cell-cell interactions and cell processes such as cell jamming and live cell extrusion are shown in Fig. 4:

Cell response to mechanical stress is a multi-time process. A timescale of about a minute corresponds to the cadherin turnover time [62] and the shape relaxation of active cells [63], while several tens of minutes correspond to the cell polarisation time [2, 64] and focal adhesion lifetime [65]. Gene expression occurs over a time scale of hours and can induce delays in the cell’s response to mechanical and biochemical stimuli. Post-translational modification of membrane proteins, such as phosphorylation and glycosylation, may only require a few minutes, whereas synthesis of proteins and their transport can take tens of minutes [66]. In our further consideration, it is necessary to discuss how various types of cell-cell interaction, pronounced in an overcrowded environment, influence biological processes such as: cell jamming and live cell extrusion.

An increase in isotropic and anisotropic parts of cell compressive stress, caused by active wetting/de-wetting of epithelial monolayers, can trigger two cell processes: cell jamming and live cell extrusion. It is well known that an increase in compressive stress intensifies cell-cell interactions. While both cell processes are extensively studied, it is not clear how cells make a decision about which of them will be favoured. The cell jamming state transition has been discussed in the context of the contact inhibition of locomotion [15, 18], while the live cell extrusion has been discussed in the context of the generation of the topological defects of cell alignment [5, 54]. Both processes result in a decrease in the undesirable compressive stress. It would be interesting to discuss both processes in the context of cell-cell interactions and to point out the physical mechanisms of cell compressive stress reduction.

### The cell jamming-to-unjamming transition and its inverse

The cell jamming state transition is caused by the contact inhibition (CIL) of locomotion, which occurs under higher cell compressive stress [15, 17, 18]. The CIL is caused by head-on cell-cell interactions resulting in cell re-polarisation and down-regulation of their propulsion forces, accompanied by weakening of cell-cell and cell matrix adhesion contacts [15–17]. The cell head-on interactions can occur during the collision of forwards and backwards flows caused by inhomogeneous wetting [18]. During a interaction, a switch in the activity of the RhoGTPases occurs at the contact site. RhoA generates contraction through the regulation of actomyosin and activation of ROCK, while Rac1 drives the formation of lamellipodia by mediating actin polymerisation. Under head-on cell interaction, RhoA is activated and Rac1 is inhibited, driving paralysis in the membrane and loss of protrusions [16]. Canales-Coutiño and Mayor [67] pointed to the role of Piezo 1 by cooperating with semaphorins in the regulation of Rac1 during the migration of neural crest cells. An increase in cell packing density, in this regime, results in a decrease in the average time between cell interactions. The average re-polarization time during the rearrangement of confluent MDCK cell monolayers is $\:1.28\:\text{h}$ [2].

When the time between two cell head-on interactions is shorter than the cell re-polarization time, cells do not have enough time to adapt to the changed micro-environmental conditions [18]. In this case, cells undergo a transition from the active (contractile) to the passive (non-contractile) state, i.e., the jamming state transition, and the cell velocity drops to zero [18]. Cells are trapped in the jamming state for a period of time and then undergo unjamming. Even though cell jamming has been well investigated, our understanding of how jamming cells control compressive stress is still in its early stages. This is linked to the interrelation between epithelial cohesion and adhesion energies.

#### Cell jamming leads to a decrease in the cell compressive stress

Active, contractile cells store more elastic energy than non-contractile ones. The contractility of epithelial cells enhances the strength of cell-cell adhesion contacts [13]. Consequently, the surface tension of active contractile cells and the cell cohesion energy, is higher than the surface tension of non-contractile (jammed) cells, i.e., $\:{\gamma\:}_{e}^{m}>{\gamma\:}_{e}^{r}$ [13]. However, the cell-cell adhesion energy of non-contractile (jammed) cells is higher than the cell-cell adhesion energy of contractile cells [19]. This is in accord with the fact that dynamical focal adhesions are needed for cell migration: too little adhesion does not provide sufficient traction, whereas too much adhesion renders the cells immobile [17, 19]. If the cell-matrix adhesion energy $\:{\omega\:}_{a}$ is higher than the cohesion energy $\:{\omega\:}_{c}$, i.e., $\:{\omega\:}_{a}>{\omega\:}_{c},$ and consequently the spreading factor $\:{S}^{e}>0$, the jamming cell domains undergo passive extension (wetting) towards surrounding migrating cells, leading to an increase in the cell packing density of the surrounding multicellular domains. Kaliman et al. [44] reported an increase in the cell packing density in homeostatic cell domains compared to jamming domains, providing experimental confirmation of this scenario. The passive extension (wetting) of jamming multicellular domains leads to a decrease in cell packing density and a decrease in cell compressive stress. Then cells undergo an unjamming transition and start migration again. The main question arising is: can cells prevent the jamming and retain active contractile states through live cell extrusion under higher cell compressive stress?

### Extrusion of live cells

Extrusion is a cellular way of reducing the cell packing density by reduced compressive stress, through the removal of a particular cell. A few conditions should be satisfied for cell exclusion to succeed: (1) the target cell must lose contact with the substrate matrix and retain its active (contractile) state and (2) the target cell must be surrounded by contractile cells, forming contractile actin rings [4]. Eisenhoffer et al. [4] and Franco et al. [68] considered live cell extrusion in MDCK monolayers and zebrafish epidermis and pointed out that stretch-activated ion channels Piezo1 influence Rho kinase (ROCK)-mediated actomyosin contractions, which are involved in the underlying mechanism of cell extrusion. Levayer et al. [69] studied live cell extrusion in the midline region of the Drosophila pupal notum and revealed that caspase 3 activation is required for cell delamination. The extruded cell stays alive for 2–4 h and then undergoes programmed death, i.e., anoikis [4]. The anoikis is caused by the loss of contact with the extracellular matrix (ECM) and exhibits some unique features in terms of cell signalling. It involves several major signalling pathways, including integrin signalling, PI3K-AkT signalling, and FAs signalling [4].

Eisenhoffer et al. [4] triggered the extrusion of live MDCK cells from the monolayer by externally induced in-plane compression of a substrate matrix. In this case, the breaking of FAs is caused by externally generated compressive stress. Saw et al. [5] revealed that the prerequisite for cell extrusion in an overcrowded environment is the formation of topological defect in cell alignment within the monolayer.

Perturbation of cell alignment can induce an inhomogeneous distribution in the strength of cell-cell adhesion contacts per single cell caused by cell glancing interactions, while cells retain their polarisation. Along this line, some cell-cell adhesion contacts are more stretched than others, leading to single-cell partial rotation in order to re-align again. This rotation causes the generation of torsional stress on cell-matrix FAs by stimulating the detachment of FAs. Consequently, we can conclude that the cell glancing interactions cause weakening of FAs, and on that basis, trigger live cell extrusion, while cell-cell head-on interactions lead to cell jamming.

Live cell extrusion frequently represents a single-cell event. However, Deforet et al. [34] and Pérez-González et al. [11] discussed the formation of 3D cell structure in the form of rim-like structure during rearrangement of epithelial monolayers in the context of collective extrusion.

## De-wetting and formation of tri-dimensional peripheral rim: the role of topological defects

Pérez-González et al. [11] observed an inhomogeneous wetting of cell monolayers such that the extension of central region is more pronounced compared to the extension of peripheral region of the monolayers. The phenomenon was discussed in the context of cell contractility. It was found that the cell contractility in the peripheral region is higher than that in the central region [11]. The increased contractility contributes to the reinforcement of E-cadherin mediated cell-cell adhesion interactions [13]. As a result, the strength of cell-cell adhesion contacts, and consequently, the epithelial surface tension, are greater in the peripheral region, while the epithelial spreading factor is lower in the central region. Intensive spreading of cells from the central region towards the peripheral region causes an increase in the cell packing density and cell compressive stress in the peripheral region [11]. The establishment of the epithelial surface tension gradient $\:\overrightarrow{\nabla\:}{\gamma\:}_{e}$ influences the generation of an interfacial tension gradient $\:\overrightarrow{\nabla\:}{\gamma\:}_{em}$ (Eq. 1) and cell shear stress $\:{\stackrel{\sim}{\varvec{\sigma\:}}}_{\varvec{e}\varvec{r}\varvec{S}}$ (Eq. 3). Cell shear stress could be the main physical factor responsible for the generation of the topological defects in cell alignment in the peripheral region. The existence of partially circular cell trajectories, as observed by Deforet et al. [33], can be a certain indicator of the presence of the cell shear stress. Cell shear stress was observed during the wetting of epithelial monolayers [1]. Despite a significant increase in cell packing density in the peripheral region of the monolayer, cells retain strong cell-cell adhesion contacts and maintain their active, contractile state [11]. The concentration of E-cadherin and consequently, the average epithelial surface tension, oscillates about some maximum value during de-wetting [11]. It means that cell glancing interactions, rather than cell head-on interactions influence the cell rearrangement in this region. As mentioned above, the cell head-on interactions would trigger the contact inhibition of locomotion associated with weakening of cell-cell and cell matrix adhesion contact and a decrease in the epithelial surface tension. However, cell glancing interactions can cause some cells to lose their FAs within the peripheral region and retain strong cell-cell adhesion contacts, resulting in their collective extrusion. It appears that dividing cells are more vulnerable to interactions between neighbouring cells and lose their FAs more quickly [34]. However, the cell divisions were significantly suppressed by the cell packing density $\:{n}_{e}\ge\:{10}^{6}\:\frac{cells}{{cm}^{2}}$ [30, 34].

**Fig. 5:**
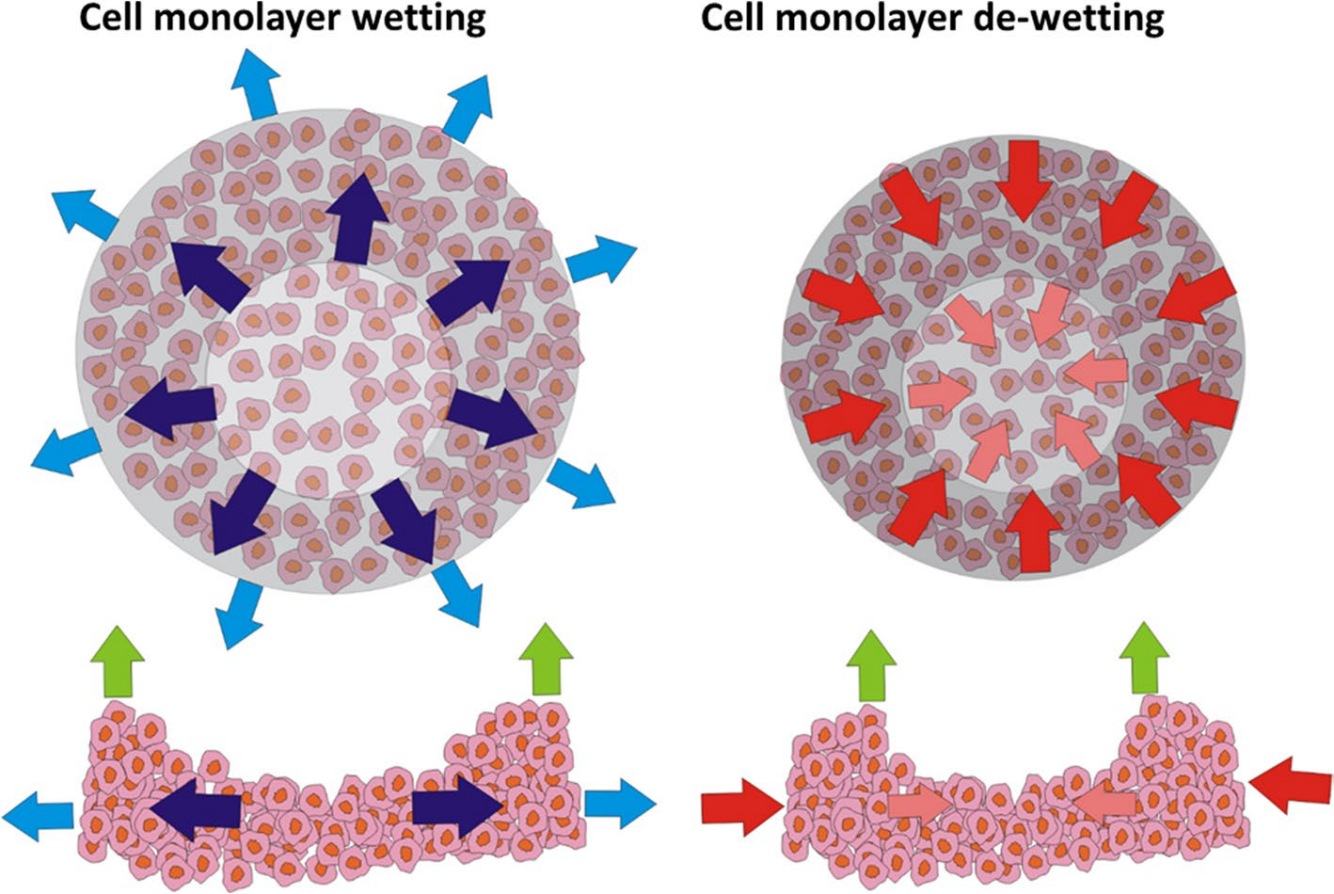
Formation of 3D rim-like structure during cell monolayer de-wetting. Blue arrows represent wetting (extension) and red arrows represent de-wetting (compression) of the monolayer, while green arrows indicate the formation of 3D rim-like structure (i.e., collective cell extrusion)

The epithelial surface tension does work on reducing the surface of the peripheral region of the monolayer, $\:{W}_{A}\left(\tau\:\right)$ given by:

4 $$\:\frac{d{W}_{A}\left(r,\tau\:\right)}{d\tau\:}=-{\gamma\:}_{e}\frac{dA}{d\tau\:}.$$

where $\:{W}_{A}\left(r,\tau\:\right)$ is the work of epithelial surface tension on cells within the peripheral region which already lost their FAs and $\:A\left(r,\tau\:\right)$ is the surface area of the peripheral region. The work $\:{W}_{A}\left(r,\tau\:\right)$ is responsible for collective cell extrusion. Collective cell extrusion in the form of 3D rim-like structure is shown in Fig. 5:

Cells within the extruded 3D rim-like structure retain their active contractile state and their polarities, in contrast to individual extrusion [34]. The phenomenon of formation of the rim-like cellular structure has also been observed for human umbilical vein endothelial cells on adherent stripes under in vivo conditions [70].

## Conclusion

This theoretical review considered physical aspects of epithelial response under high compressive stress caused by collective cell migration. Higher cell compressive stress (a few hundreds of Pa) characteristic for high cell packing density ($\:\ge\:{10}^{6}\:\frac{\text{cells}}{{\textrm{cm}}^{2}}$) causes intensive cell-cell interactions, which can perturb cell alignment. Two scenarios can arise as a result of these interactions: (1) the cell jamming state transition and (2) live cell extrusion. However, it has not been clear how cells make a decision about whether to undergo jamming or live cell extrusion. Both phenomena have been observed experimentally during the wetting/de-wetting of epithelial monolayers. The main results were obtained by discussing the dynamics of epithelial monolayer wetting/de-wetting by emphasizing the physical aspects of cell-cell interactions obtained on various cell monolayers. We can summarize them as follows:

- The main characteristics of cell rearrangement during epithelial wetting/de-wetting are: (1) the anisotropic nature of collective cell migration; and (2) the inhomogeneous distribution of physical parameters such as cell packing density, cell velocity, cell mechanical stress, cell tractions, epithelial surface tension, epithelial-matrix interfacial tension, and their oscillatory changes over a time scale of hours. Consequently, the epithelial monolayers can be treated as ensembles of multicellular domains characterized by homogeneous distributions of these physical parameters per domain.
- The cell compressive stress component, as well as tensional and shear stress components, are generated locally during wetting and de-wetting and influences the active and passive displacement of multicellular domains.
- In addition to the cell compressive stress, the cell alignment perturbation is affected by the gradient of cell-matrix interfacial tension, which is a component of the cell shear stress and is addressed in relation to topological defects.
- Two types of cell-cell interactions can be distinguished: cell head-on interactions and glancing interactions. While cell head-on interactions are induced primarily by the collision of cell forwards and backwards flows, glancing interactions can be induced by the interplay between compressive and shear stress components.
- The cell head-on interactions cause cell re-polarisation leading weakening of cell-cell and cell-matrix adhesion contacts. When the time between two-cell head-on interactions is shorter than the cell re-polarisation time, cells undergo the jamming state transition (i.e., the contractile-to-non contractile cell state transition).
- The cell glancing interactions are not strong enough to induce cell re-polarisation, but can perturb cell alignment. Consequently, cells retain their polarisation accompanied by strong E-cadherin-mediated adhesion contacts. The altered perturbation of cell alignment causes an inhomogeneous distribution of the strength of cell-cell adhesion contacts per individual cell. It is in accord with the fact that some adhesion contacts are stretched while others are compressed. This inhomogeneous distribution of the strengths of cell-cell adhesion contacts can induce single-cell rotation, resulting in the generation of torsional shear strain on focal adhesions, which leading to their detachment from the substrate matrix. This particular cell can then be extruded from the monolayer.
- Live cell extrusion from overcrowded regions of the cell monolayer can be a collective phenomenon, inducing the formation of a 3D cell rim-like cell structure on the monolayer.

Additional experiments are needed to examine the impact of cell glancing interactions on the state of cell-matrix focal adhesions.

## Data Availability

Not applicable.

## Data availability

No datasets were generated or analysed during the current study.
